# Imaging in pelvic exenteration—a multidisciplinary practice guide from the ESGAR-SAR-ESUR-PelvEx collaborative group

**DOI:** 10.1007/s00330-024-10940-z

**Published:** 2024-08-25

**Authors:** Stephanie Nougaret, Doenja M. J. Lambregts, Geerard L. Beets, Regina G. H. Beets-Tan, Lennart Blomqvist, David Burling, Quentin Denost, Maria A. Gambacorta, Benedetta Gui, Ann Klopp, Yulia Lakhman, Kate E. Maturen, Riccardo Manfredi, Iva Petkovska, Luca Russo, Atul B. Shinagare, James A. Stephenson, Damian Tolan, Aradhana M. Venkatesan, Aaron J. Quyn, Rosemarie Forstner

**Affiliations:** 1 Department of Radiology, PINKCC lab, U1194, Montpellier Cancer Center, Montpellier, France; 2 Department of Radiology, Netherlands Cancer Institute, Amsterdam, The Netherlands; 3 Department of Surgery, Netherlands Cancer Institute, Amsterdam, The Netherlands; 4 Department of Molecular Medicine and Surgery, Karolinska Institutet, Sweden & Department of Radiation Physics/Nuclear Medicine, Karolinska University Hospital, Solna, Sweden; 5 Intestinal Imaging Centre, St Mark’s Hospital, London North West University Healthcare NHS, London, UK; 6 Bordeaux ColoRectal institute, Clinique Tivoli, Bordeaux, France; 7 Department of Radiation Oncology, Fondazione Policlinico Universitario A.Gemelli IRCCS, Rome, Italy; 8 Department of Bioimaging, Radiation Oncology and Hematology, Fondazione Policlinico Universitario Agostino Gemelli IRCCS, Rome, Italy; 9 Department of Radiation Oncology, University of Texas M.D. Anderson Cancer Center, Houston, TX USA; 10 Department of Radiology, Memorial Sloan Kettering Cancer Center, New York, NY USA; 11 Departments of Radiology and Obstetrics and Gynecology, University of Michigan, Ann Arbor, MI USA; 12 Department of Bioimaging, Radiation Oncology and Hematology, UOC of Radiodiagnostica Presidio Columbus, Fondazione Policlinico Universitario A. Gemelli IRCSS, Rome, Italy; 13 Department of Radiology, Brigham and Women’s Hospital, Dana-Farber Cancer Institute, Harvard Medical School, Boston, MA USA; 14 Department of Radiology, University College London Hospitals NHS Foundation Trust, London, UK; 15 Department of Radiology, St James’s University Hospital, Leeds, UK; 16 Department of Abdominal Imaging, Division of Diagnostic Imaging, The University of Texas M.D. Anderson Cancer Center, Houston, TX USA; 17 John Goligher Colorectal Unit, Leeds Teaching Hospitals NHS Trust, Leeds, UK; 18 Department of Radiology, Uniklinikum Salzburg, PMU, Salzburg, Austria

**Keywords:** CT, MRI, PET/CT, Pelvic exenteration, Cancer

## Abstract

**Abstract:**

Pelvic exenteration (PE) is a radical surgical approach designed for the curative treatment of advanced pelvic malignancies, requiring en-bloc resection of multiple pelvic organs. While the procedure is radical, it has shown promise in enhancing long-term survival and is now comparable in surgical mortality to elective resections for primary pelvic cancers. Imaging plays a crucial role in preoperative planning, with MRI, CT, and PET/CT being pivotal in assessing the extent of cancer and formulating a surgical roadmap. This paper presents clinical practice guidelines for imaging in the context of PE, developed jointly by ESGAR, SAR, ESUR, and the PelvEx Collaborative. These guidelines aim to standardize imaging protocols and reporting to improve the preoperative assessment and facilitate decision-making in the multidisciplinary treatment of pelvic cancers. Our recommendations underscore the importance of a multidisciplinary approach and the need for clear and precise imaging reports to optimize patient care.

**Clinical relevance statement:**

Our recommendations underscore the importance of a multidisciplinary approach and the need for clear and precise imaging reports to optimize patient care.

**Key Points:**

- *MRI is mandatory for local staging in pelvic exenteration*.
- *Structured reporting (using the template provided in this guide) is recommended*.
- *Multidisciplinary review of imaging is critical for surgical planning*.

## Introduction

Pelvic exenteration (PE) is a highly complex surgical procedure that is primarily used to treat locally advanced and recurrent pelvic cancers. This may include gastrointestinal, genitourinary, gynecologic, and occasionally musculoskeletal malignancies [1–5]. During this procedure, at least two pelvic organs are removed en-bloc, followed by reconstruction and/or diversion of bowel/urinary/sexual functions. The primary objective of surgery is to excise cancer with curative intent (i.e., complete cancer resection with negative surgical margins, R0), often necessitating the removal of adjacent or involved organs, bones, muscles, nerves, and vascular structures. These extensive resections are associated with significant morbidity and are rarely performed for palliation of symptoms alone [1–5]. The multidisciplinary team (MDT) is critical for patient selection to help optimize patients’ quality of life and survival. Magnetic resonance imaging (MRI), computed tomography, (CT), and positron emission tomography/computed tomography (PET/CT) are the most commonly used imaging modalities for the assessment of cancer extent and for planning an imaging roadmap to inform subsequent surgery (what to remove and what to leave behind) [6, 7]. MRI is the modality of choice for the accurate localization and characterization of disease in the pelvis [6, 8–10]. CT is the first-line modality for distant staging. PET/CT is often used as a complementary tool to assess CT occult disease, particularly lymph nodes, and to define the metabolic activity and extent of the local tumor in the pelvis [9, 11].

Despite the importance of this topic, literature on PE imaging and radiologic reporting is limited [6, 12]. Given the complexity of this surgical procedure, preoperative radiology reports should be structured, clear, and relevant. The objective of this joint paper from the European Society of Gastrointestinal and Abdominal Radiology (ESGAR), Society of Abdominal Radiology (SAR), European Society for Urogenital Radiology (ESUR) and PelvEx collaborative is to propose clinical practice guidelines for imaging in the setting of PE. Our goal is to provide a practical resource for radiologists, including tips for imaging protocol development and reporting.

## Methods

### Panel selection

The ESGAR research committee initiated this practice guide and appointed two radiologists, S.N. and D.L., as the chairs. The chairs then composed the multidisciplinary panel, which included representatives from ESGAR (D.L., S.N., R.BT., J.S., L.B., D.T.), SAR (I.P., Y.L., K.M., A.S., A.V.), ESUR (R.F., S.N., B.G., R.M., L.R.), and the PelvEx Collaborative (A.Q., G.B., D.B., Q.D.), all of whom are specialists working in dedicated referral centers performing PE. The panel also included two radiation oncologists (A.K. and M.A.G.) as independent clinical members, both recognized for their expertise in the field. A fellow (L.R.) was appointed to review the literature. The selection criteria for the panel emphasized prior peer-reviewed publications in the field and broad geographical representation. In total, the panel consisted of 21 members, who are the authors of this article.

### Working groups

The multidisciplinary expert panel was divided into three working groups, each assisted by 1–2 senior deputies:

1. Principles of clinical practice (A.Q., A.K., G.B., Q.D., M.A.G.; deputy A.Q.): This working group reviewed the existing literature, compiling a narrative review centered on surgical and neoadjuvant treatment options.
2. Acquisition protocol (R.F., L.B., I.P., D.B., R.M., J.S., R.B.-T.; deputy R.F.): This working group developed recommendations for the MR protocol and quality standards. Their recommendations were informed by the results of an online questionnaire crafted by RF and distributed to all group members. A majority consensus of at least 80% of the members was considered the benchmark for inclusion.
3. Reporting template (S.N., D.L., B.G., A.V., A.S., K.M., Y.L., D.T.; deputies S.N., D.L.): This working group formulated recommendations for image interpretation and reporting. They designed a structured reporting template informed by an online questionnaire developed by D.L. and S.N., which was shared with all working group members and surgical representatives (A.Q. and G.B.).

### Stepwise process

Initially, L.R. conducted a systematic review of the literature to establish the current state of evidence for the topics addressed by various working groups. A search was performed in November 2022 using the United States National Library of Medicine PubMed journal citation database. The search string (detailed in Appendix A) focused on imaging, did not have date limits, and yielded 19 publications. This included eleven original articles, seven reviews, and one meta-analysis. An additional search was then undertaken targeting literature not specifically tied to imaging (refer to Appendix A for the search string). This search produced 20 additional publications, including 13 original articles and seven reviews.

All retrieved literature was accessible to the members of each working group and served as the basis for drafting recommendations and designing the questionnaires. Each working group held several meetings. After these discussions, the preliminary input and recommendations from each group were forwarded to the panel chairs (S.N., D.L.) who produced a draft manuscript which was circulated to the full panel. A final meeting with the full panel on October 19, 2023 finalized the recommendations.

## Principles of clinical practice

Considerable progress has been made in the management of patients with locally advanced or recurrent cancers of the pelvis since the inception of PE. Generally considered incurable or technically inoperable, these complex patients were historically treated with palliative chemoradiation, with many patients experiencing intractable symptoms. In contrast, the surgical mortality from PE is now comparable to that of elective resection for primary pelvic cancers [2–4]. A complete resection with negative surgical margins (R0), i.e. clear of both macroscopic and microscopic tumor involvement, is well-established as a critical prognostic factor, associated with both local control and long-term survival [13–16].

The operative challenge for PE stems from extension of cancer and associated fibrosis (due to leak, perforation, or treatment) beyond standard anatomical planes for resection [17–19]. Multidisciplinary surgical collaboration is frequently necessary and may include urologic, vascular, orthopedic, and plastic surgeons. In contrast to conventional surgical procedures (such as total mesorectal excision for colorectal cancer) whereby dissection proceeds along developmental tissue planes, PE utilizes a radical surgical approach that can include multiple pelvic compartments for en-bloc excision [20–23]. A detailed description of these surgical approaches is beyond the scope of this article. Nevertheless, it is important to be aware that previously accepted limitations of the extent of PE have now been challenged. High sacrectomy, extended pelvic sidewall clearance including the sciatic foramen, resection, and reconstruction of common and external iliac vessels are well described [16, 24]. Moreover, these advanced resections are now supported with evidence demonstrating acceptable morbidity, high rates of R0 resection, and preserved quality of life. Today, PE is established for locally advanced or locally recurrent rectal cancers as well as the treatment of choice in patients with advanced malignancies arising from other pelvic organs, including recurrent gynecologic or urologic tumors, pelvic soft tissue malignancies, and tumors of the bony pelvis.

Prior to PE, neoadjuvant therapy may be considered to reduce tumor volume, control disease, and facilitate surgical removal [25]. Response to neoadjuvant therapy depend on the pelvic malignancy histologic type. As such, these decisions must be guided by oncologists with appropriate expertise who may use a combination of radiotherapy, chemotherapy, or targeted therapy [26].

While neoadjuvant therapy appears to have oncological advantages, it may have disadvantages in some individuals including multiorgan deconditioning and disease progression (locally or systemically) during the course of treatment. In addition, tumor regression-related fibrosis and inflammation can increase the morbidity of subsequent surgery. The recent findings from a multinational cohort of 1291 patients who underwent PE revealed that while 78% underwent some form of neoadjuvant therapy (64% chemoradiotherapy; 10% radiotherapy alone; 3% chemotherapy alone; 23% treatment details unknown), this did not translate to a statistically significant benefit in survival [27]. For now, there is a lack of standardized recommendations for the follow-up of patients after PE, highlighting a valuable area for future guideline development.

## Recommendations for image acquisition

Detailed recommendations can be found in Table 1; key points are summarized below:

**Table 1:** Summary of recommendations^a^ for image acquisition in pelvic exenteration (PE)

Preoperative imaging before PE includes:	**Key modalities:** • MRI of the pelvis • [^18^F]FDG PET/CT obtained from skull base to mid-thigh • CT of the chest, abdomen and pelvis acquired during the portal-venous contrast phase **Complementary (optional) modalities:** • Liver MRI, in particular in patients with high-risk disease (for example macroscopic lymphovascular disease). • Abdominal or whole-body MRI
MRI (1.5 T or 3.0 T)	**Strengths:** • Detailed anatomical assessment of local tumor extent • Differentiation between tumor and fibrosis (DWI) **Coverage:** Pelvis **Preparation:** No consensus was achieved regarding fasting before the MRI, urinary bladder filling, antispasmodic agent use, or rectal cleansing. **Protocol (Sequences):** • T2WI in three planes (slice thickness 3–4 mm), typically sagittal and two additional planes. Oblique planes are performed for local staging of rectal, uterine and vaginal masses. Rationale: Determine maximal tumor size, extent, and relationship to the pelvic sidewall. • DWI (slice thickness 4–5 mm): typically in line with T2W in (oblique-)axial plane. High *b*-value of > 800–1000 mm/s^2^ with ADC maps (visual assessment only). Rationale: Adjunct to T2WI to determine maximal tumor size, extent and relationship to the pelvic sidewall; distinguish tumor from fibrosis, facilitate lymph node detection. • Post contrast(Gd) T1WI: with a dynamic or a multiphase technique. Rationale: Adjunct to T2WI/DWI to determine tumor extent, assess vascularization, lymph nodes • (Optional:) Non fat-supressed axial T1WI (agreement 75%). Rationale: helps to delineate pelvic anatomy including the pelvic sidewall and bone infiltration
[^18^F]FDG PET/CT	**Strengths:** • Detection of lymph node metastases, in particular pelvic sidewall and (unenlarged) retroperitoneal nodes • Detection of (small) multifocal pelvic disease • Detection of extrapelvic disease **Coverage:** Skull base to mid-thigh **Preparation:** Fasting 4–6 h **Protocol:** IV contrast optional
CT	**Strengths:** • Vascular assessment • Detection of distant disease **Coverage:** chest, abdomen and pelvis **Preparation:** No consensus on oral contrast was achieved (used by 61% of panel, majority preferring positive oral contrast) **Protocol:** imaging in portal-venous phase with multiplanar reconstructions

^a^ Note, unless otherwise specified, recommendations are based on > 80% consensus among panelists, based on a questionnaire distributed among 14 specialists in imaging and oncology. All recommendations were subsequently discussed and approved by the full panel)

### Quality criteria

Given the adverse impact of disease progression and the significant resources invested in PE, imaging should be conducted shortly prior to PE to limit the risk of interval local and distant disease progression. There are no strict guidelines about the permissible time interval and the panel acknowledges that it is in part determined by tumor biology (e.g. growth rate on serial imaging exams). As a rule of thumb, an interval of 30 days between staging scans (CT and MRI) and PE should be considered optimal. However, the authors acknowledge, that this interval might be lengthened (up to 60 days) in specific cases for example where the disease has responded well to neoadjuvant treatment and has been unchanged on serial imaging and where clinical evaluation and laboratory findings do not suggest progressive disease. Every PE candidate should be discussed in a specialized multidisciplinary tumor board close to the date of PE.

### Imaging techniques and coverage

#### Before pelvic exenteration

Though the choice of which (combination of) imaging modalities to use depends on the clinical scenario and should be discussed in a multidisciplinary setting, the panel unanimously agreed that an MRI of the pelvis is mandatory for local staging and to provide a surgical roadmap prior to PE. CT and [^18^F] PET/CT have complementary values for local staging and are used to screen for distant disease sites. The respective benefits of MRI, CT, and PET/CT are listed in Table 1 [9, 11, 28–30].

Possible additional procedures include liver MRI with liver-specific contrast agents, or abdominal or whole-body MRI [31]. Owing to limited access, hybrid imaging using PET/MRI (or MRI and PET fusion) is currently not the standard of care. Integrated reporting of PET and diagnostic CT with i.v. contrast by a hybrid imaging specialist or team of radiologists and nuclear medicine specialists can offer complementary value but is not standard practice.

#### For post-operative assessment of complications

The nature of the suspected complications dictates the preferred imaging approach. Typically, contrast-enhanced CT is the first-choice imaging modality in both acute and chronic settings following PE. CT urography is employed to evaluate for suspected urinary leakage, whereas MRI serves as a problem-solving tool, mostly in the chronic setting [9, 32].

### Specific imaging techniques

#### MRI

Both 1.5-T and 3-T MRIs may be utilized. High-resolution T2-weighted sequences are recommended. The use of diffusion-weighted imaging (DWI) and post-gadolinium T1-weighted sequences is recommended by the majority of authors of this guideline but their use varies across countries. Trained technologists, with radiologist’s input, should adjust the field of view and sequence acquisition angle to ensure adequate anatomic coverage of the tumor, its related lymph node drainage, and adjacent pelvic structures. Of the survey respondents, 62% preferred patients to fast for 4–6 h before MRI, and 70% routinely utilized antiperistaltic agents, e.g. Buscopan® or Glucagon® to reduce motion artifacts. No consensus was reached on the recommended degree of bladder filling. These recommendations are in line with a previous guideline from the European Society of Urogenital Radiology (ESUR) and a study from Sheik-Sarraf et al who showed that fasting, antispasmodic agents, and saturation bands improved MR image quality and are recommended for female pelvic MRI while urinary bladder filling influenced the cervix-to-uterine angle, but had no clear effect on image quality [33–38]. Seventy-five percent of panelists who participated in the questionnaire administered a micro-enema for rectal cleansing prior to the examination in colorectal cancer patients, although the panel also acknowledges that the feasibility and benefits are dependent on the specific clinical setting (e.g. contraindicated after previous resection of the anal canal or in patients with low obstructing and/or painful tumors).

#### [^18^F] PET/CT

Typically, PET/CT scan extends from the skull base to the mid-thigh, contingent on tumor type. Whether to perform PET/CT with or without the addition of intravenous contrast depends on local policy with significant variations noted (also among the panelists) across different countries and institutions.

#### CT

CT scans should routinely cover the chest, abdomen, and pelvis and should be acquired during the portal-venous phase. This can optionally be augmented with either an arterial or delayed phase. While no definite recommendation exists about the use of oral contrast, 61% of the panelists who participated in the questionnaire use it, with the majority opting for a positive contrast agent. 3D processing for vascular visualization is optional and CT datasets may also be employed intraoperatively for navigation.

## Recommendations for image interpretation and reporting

### Reporting template

**Fig. 1:**
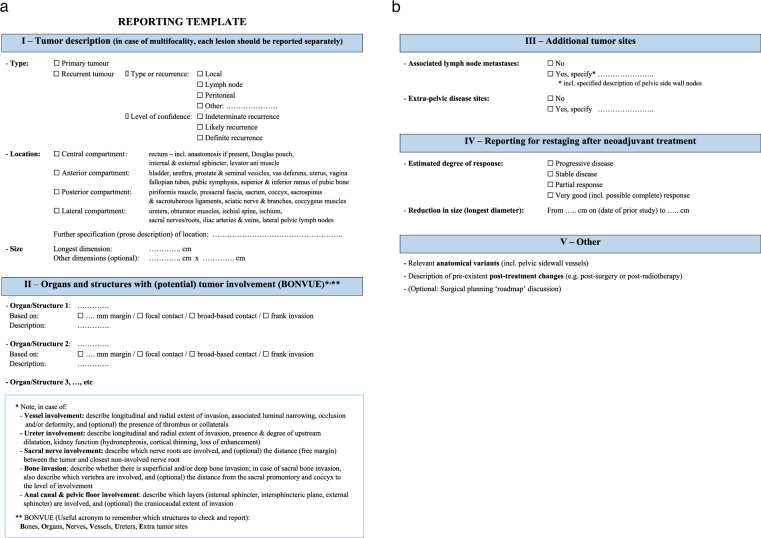
Reporting template

The panel unanimously agreed that the use of structured reporting is recommended when reporting MRI (or other imaging modalities) prior to PE. An example template can be viewed in Fig. 1 (refer to S1 to download the template).

A summary of essential recommendations on image interpretation and reporting is presented in Table 2.

**Table 2:** Key recommendations^a^ for image interpretation and reporting

How to assess organ and structure involvement	• Loss of a separating fat plane between the tumor and adjacent organ or structure should be considered a sign suspicious for involvement. • When assessing potential involvement one should discern a close margin (… mm) or focal contact from broad-based contact and frank invasion.
Anal canal and pelvic floor involvement	• Describe which layers (internal sphincter, intersphincteric plane, external sphincter) are involved. • (Optional: describe the craniocaudal extent of invasion)
Vessel and ureter involvement	• Describe the longitudinal and radial extent of invasion. • Describe associated luminal narrowing or occlusion including signs of upstream dilatation and, in cases of vessel involvement, the presence of thrombus or collaterals. • In case of ureteric involvement, describe associated signs associated with loss of kidney function (hydronephrosis, delayed nephrogram, parenchymal thinning).
Nerve involvement	• Specify the level of involvement, including a detailed description of which nerve roots are involved. • Compared with the contralateral side; asymmetry in signal, enhancement, or thickening are signs suspicious for involvement. • (Optional: Describe the distance (free margin) between the tumor and the closest non-involved nerve root).
Sacral Vertebral and other bone involvement	• Specify the level of involvement in the case of sacral vertebral involvement. • Distinguish superficial involvement (cortical irregularity or thinning) from deep bone invasion (low T1W signal). • (Optional: describe the distance from the sacral promontory and coccyx to the level of involvement).

^a^ Note, recommendations are derived from the outcome of a questionnaire that was sent out to the ‘reporting template’ working group members and clinical representatives

Debate arose among the panelists regarding the optimal way to detail organ and structural involvement within the template. Three methodologies were proposed.

A. List only those organs and structures that are, or might be, involved.
B. Provide a selective itemized list of all organs within the implicated compartment(s), specifying their involvement status.
C. Provide a comprehensive itemized list of all pelvic organs, specifying their involvement status.

The panel’s majority favored option A, which was therefore integrated into the proposed template (Fig. 1). Option B is recognized as a viable alternative. Institutions are advised to choose between Options A and B based on the preferences of their local multidisciplinary teams. A universal sentiment within the panel was the redundancy of routinely listing all the pelvic organs and structures.

**Fig. 2:**
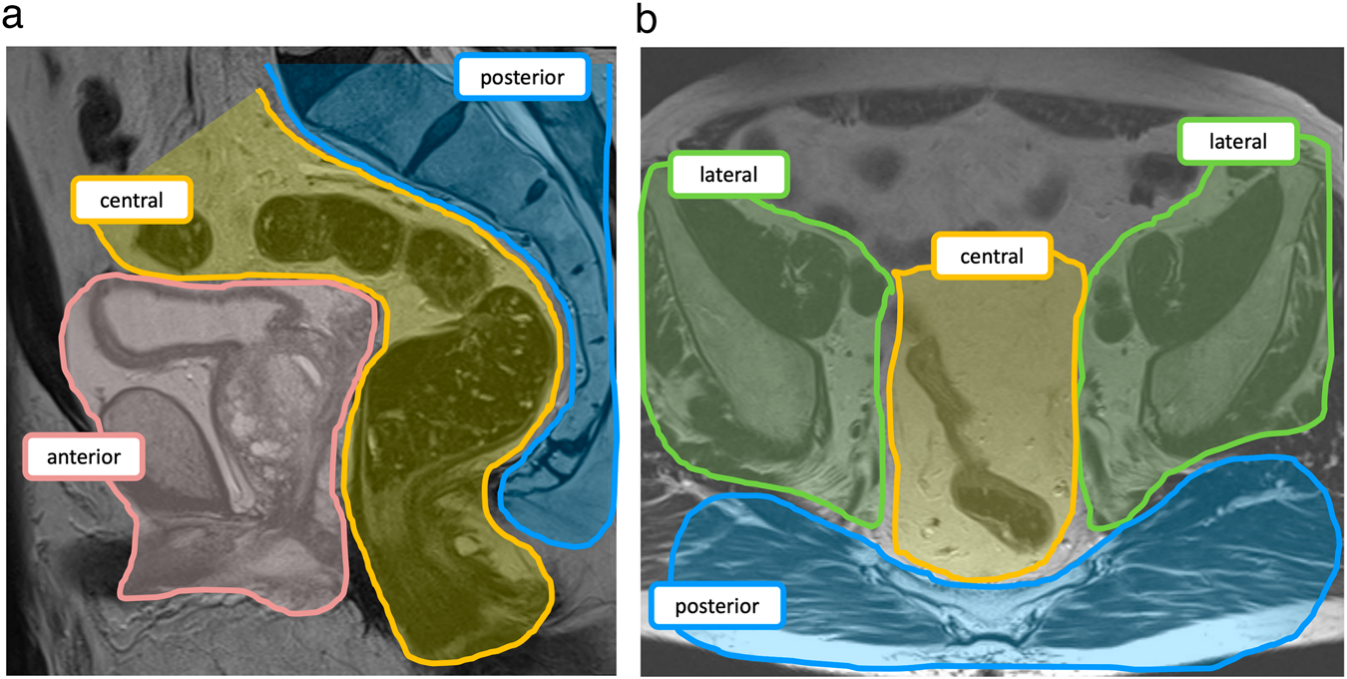
Compartment approach to describing pelvic patterns of disease in sagittal (**a**) and axial (**b**). The central or axial compartment (yellow) includes the rectum—incl. anastomosis if present, mesorectum, pouch of Douglas, internal & external sphincter, and levator ani. The anterior compartment (pink) includes the genitourinary tract, pubic symphysis, and superior & inferior rami of the pubic bone. The posterior compartment (blue) includes the piriformis muscles, presacral fascia, sacrum, coccyx, sacrospinus & sacrotuberous ligaments, sciatic nerve & branches, and coccygeus muscles. The lateral compartment (green) includes the ureters, obturator muscles, ischial spine, ischium, sacral nerves/roots, lateral pelvic lymph nodes, and iliac arteries & veins

**Fig. 3:**
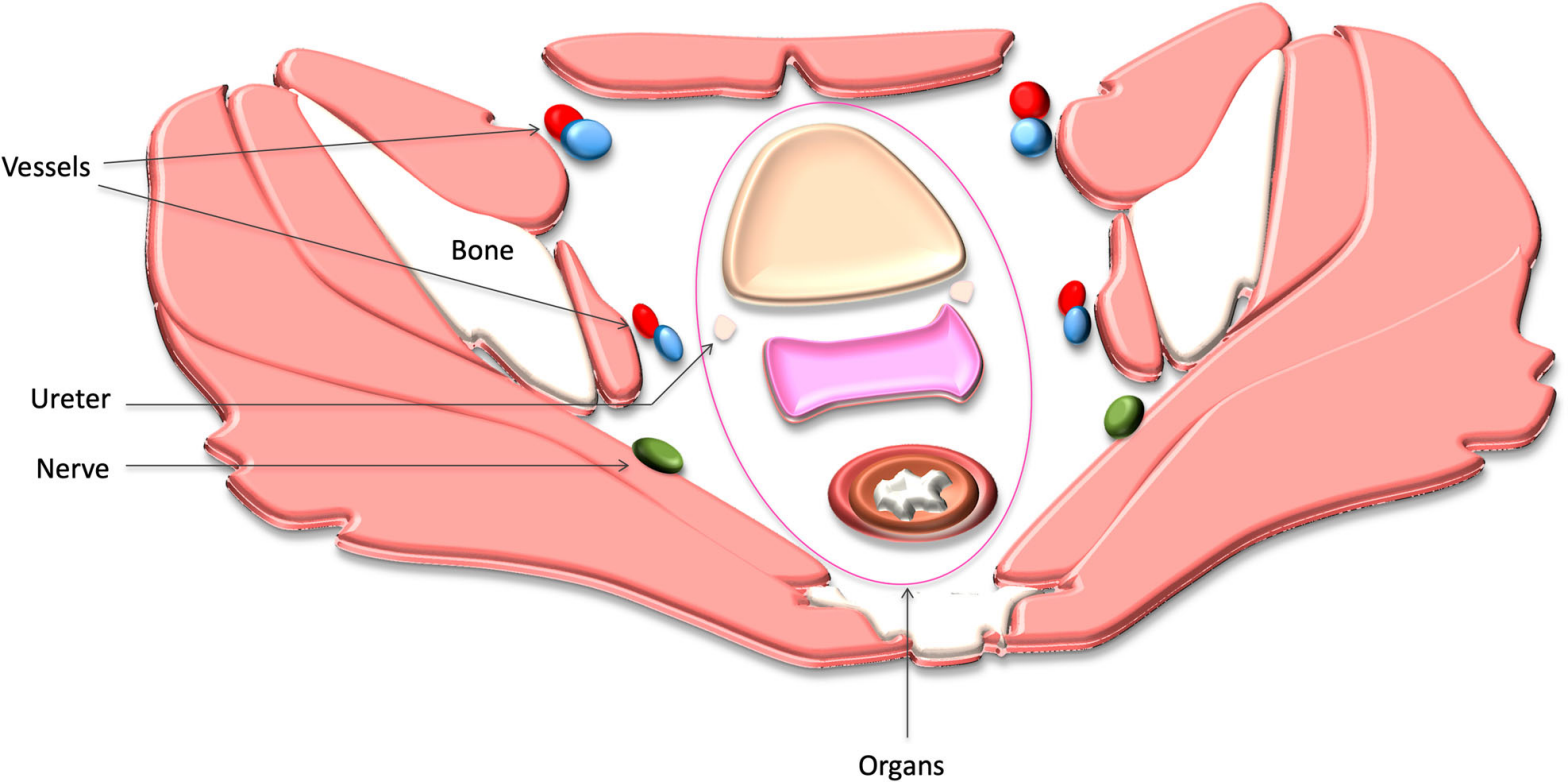
Illustration highlighting the different important structures to evaluate on imaging during preoperative assessment for pelvic exenteration using the BONVUE acronym (Bones, Organs, Nerves, Vessels, Ureters, Extra tumor sites)

**Fig. 4:**
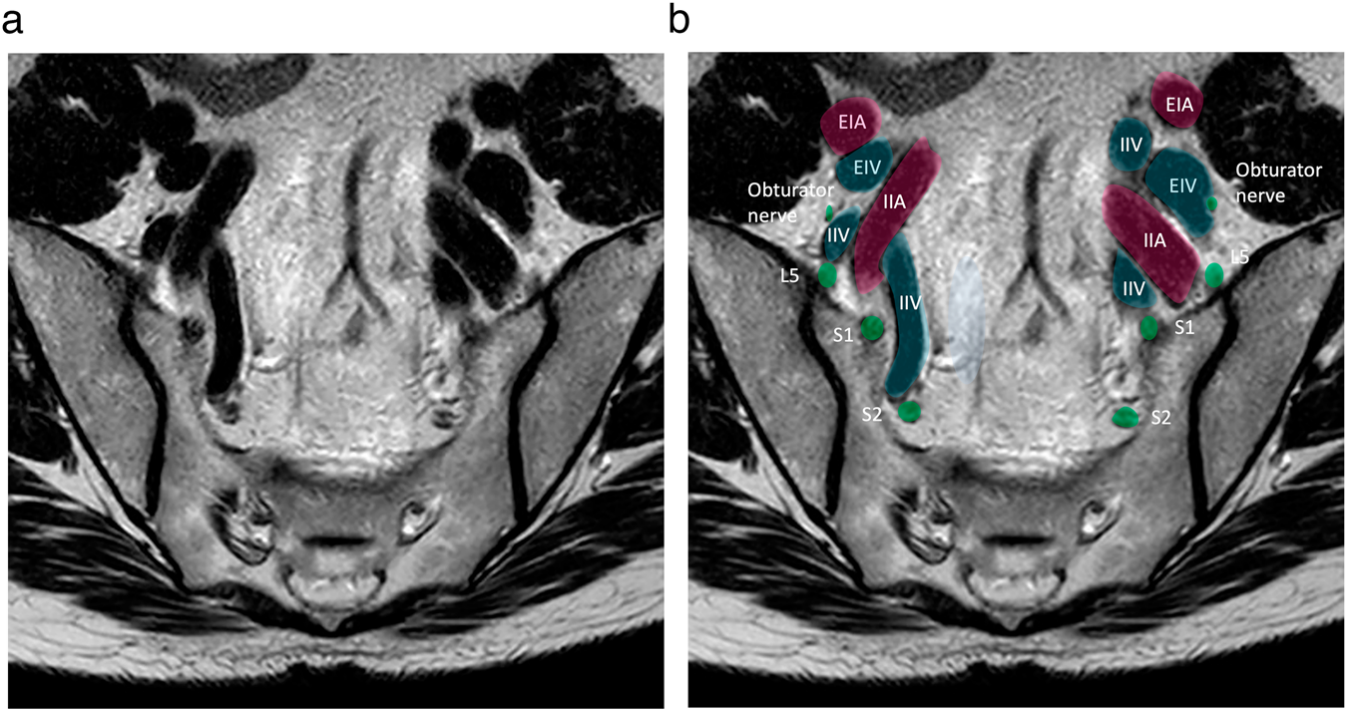
Axial T2-weighted MR image (**a** without annotations, **b** with annotations) at the level of the S2 highlighting the position of the nerves and vessels. EIA, external iliac artery; EIV, external iliac vein; IIA, internal iliac artery; IIV, internal iliac vein

**Fig. 5:**
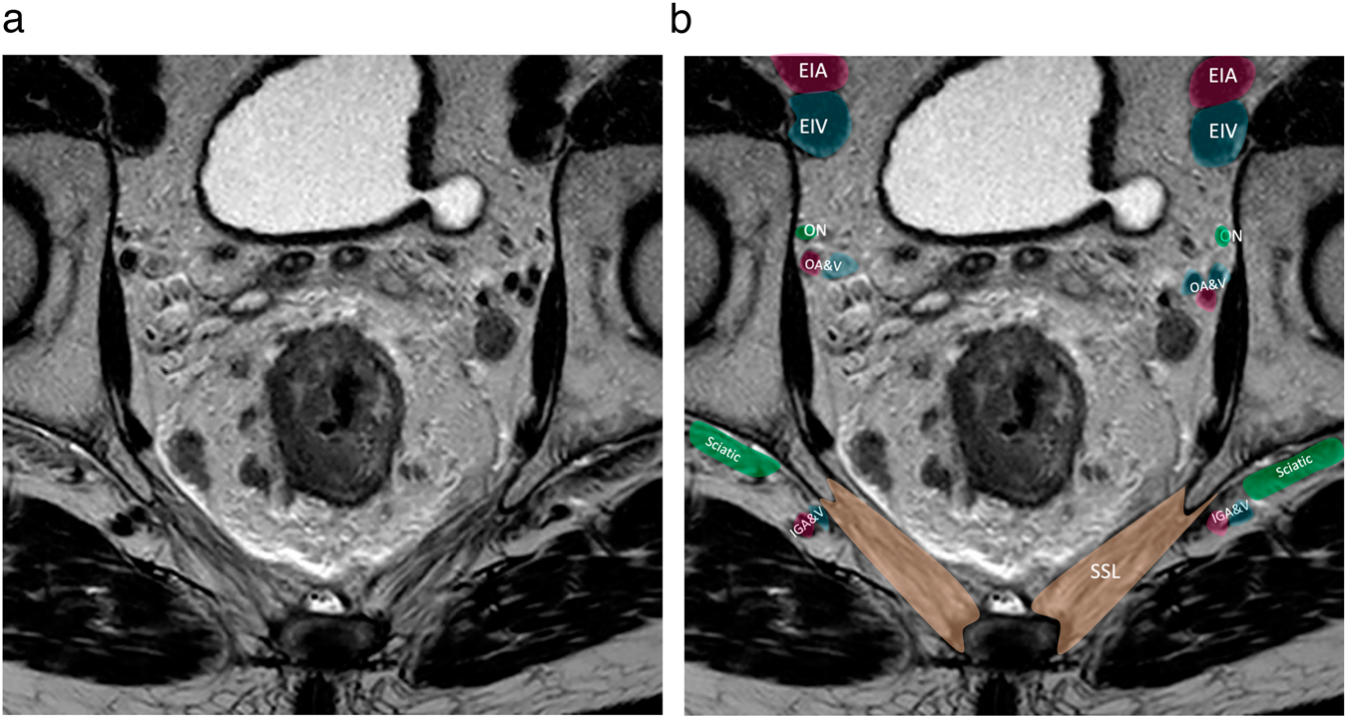
Axial T2-weighted MR image (**a** without annotations, **b** with annotations) at the level of the S5, pointing out the position of the obturator nerve which is a very good surgical landmark, especially for lymphadenectomy. SSL, sacrospinous ligament; IGA&V, inferior gluteal artery and vein; OA&V, obturator artery and vein, ON, obturator nerve; EIA, external iliac artery; EIV, external iliac vein

The acronym ‘BONVUE’ (Bones, Organs, Nerves, Vessels, Ureters, Extra tumor sites) might serve as a convenient reminder for Radiologists to include relevant items to include in reports. Many of this guideline’s authors also advocate for a compartment-centric approach. Figure 2 highlights the different pelvic compartments while Figs. 3–5 show the important structures which need to be reported.

### Restaging after neoadjuvant treatment

Included within the reporting template is a dedicated section offering guidance on reporting responses to neoadjuvant treatment. The panel advises classifying the responses using commonly used response categories such as progressive disease, stable disease, and partial response (in line with RECIST) [39]. Very good and possible complete responses are grouped together in the template as the panel acknowledges that MRI cannot accurately discriminate between a near-complete and complete response to neoadjuvant therapy in the setting of PE because of the difficulties in differentiating residual tumor from post-treatment fibrosis. DWI has shown value to improve the accuracy of MRI to identify complete responders, in particular when restaging primary rectal cancers after neoadjuvant treatment [40–42] The absence of a high signal on high *b*-value DWI is a sign suggestive of a complete response, while the presence of focal mass-like or scattered foci of high DWI signal are signs associated with the residual tumor. The value of DWI to diagnose a complete response after neoadjuvant treatment in recurrent rectal and gynecological tumors is less well-established and typically more challenging, especially in the case of more diffuse infiltrative) tumors. Any new findings, whether related to disease progression (e.g., development of new metastases), or complications caused by either disease progression or fibrosis (e.g., ureter obstruction, perforation, abscess, etc) should be emphasized in the report.

### Radiology roadmap for surgical planning

In some PE centers, it is common practice to include a documented ‘roadmap’ in the radiology report detailing the proposed surgical planning required to achieve a radical (R0) resection. This approach reflects the strong multidisciplinary collaboration required between radiologists and surgeons to combine the detailed knowledge of the anatomical extent of disease and the necessary understanding of the way that the operations are performed.

The panel agreed that the use of annotated key images (and the optional use of bookmarks or hyperlinks to link these key images to the report text) are helpful in guiding MDT discussions and informing surgical planning. The panel emphasizes that the decision on whether a patient should proceed with PE surgery and which operation to undertake is obviously not made by the radiologist. Rather, the radiology report should provide guidance to the MDT on the extent of cancer and assist in defining the technical feasibility of its R0 excision.

**Fig. 6:**
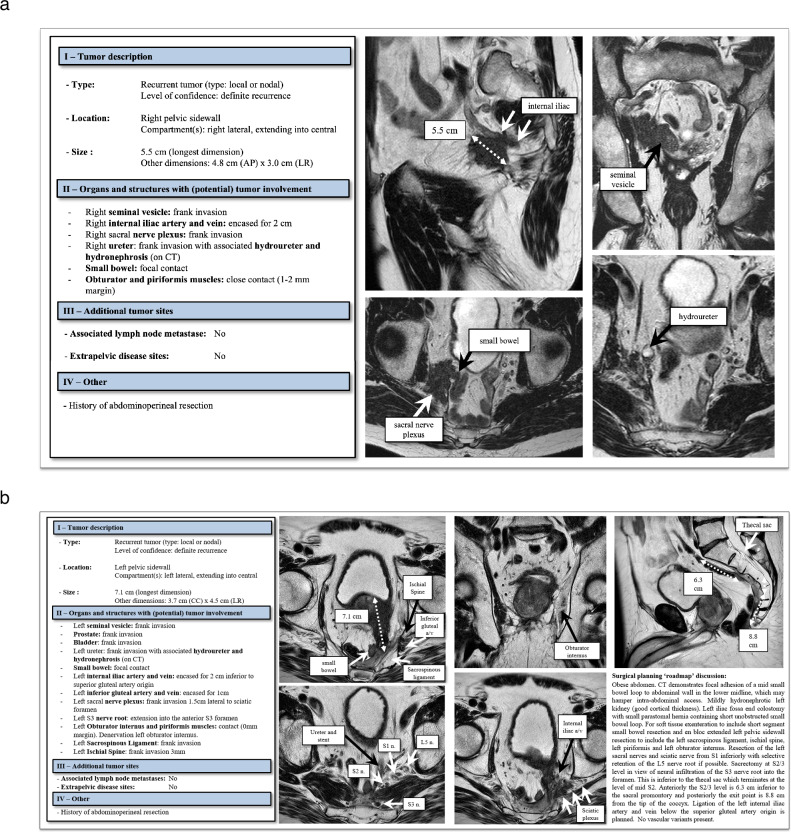
**a** Template report for a recurrent rectal tumor involving the right lateral and central pelvic compartments following prior abdominoperineal resection. **b** Template report including surgical planning ‘roadmap’ discussion for a large recurrent rectal tumor involving the central and left lateral pelvic compartments following prior abdominoperineal resection

Finally, Fig. 6a, b show clinical examples of two PE candidates with completed template reports and corresponding key images. Figure 6b also includes a surgical planning discussion as an example of how radiologists’ reports may assist surgical planning in a dedicated PE center.

## Conclusion

The ESGAR-SAR-ESUR-PelvEx collaborative group has produced the first international clinical practice guidelines for PE imaging. These guidelines emphasize the vital role of multidisciplinary collaboration and state-of-the-art imaging with MRI, CT, and PET/CT for detailed local and distant staging of the disease, and the importance of structured reporting. PE is a highly complex surgical procedure and requires the highest quality imaging and reporting standards to ensure optimal patient selection and outcomes of surgery.

## Supplementary information

Appendix A

S1

## Abbreviations

[^18^F]: Fluorine-18 (as used in PET/CT imaging)
CT: Computed tomography
DWI: Diffusion-weighted imaging
ESGAR: European Society of Gastrointestinal and Abdominal Radiology
ESUR: European Society for Urogenital Radiology
MRI: Magnetic resonance imaging
PE: Pelvic exenteration
PET/CT: Positron emission tomography/computed tomography
R0: Complete cancer resection with negative surgical margins
SAR: Society of Abdominal Radiology
