# Correction: Imaging in pelvic exenteration—a multidisciplinary practice guide from the ESGAR-SAR-ESUR-PelvEx collaborative group

**DOI:** 10.1007/s00330-025-11437-z

**Published:** 2025-03-10

**Authors:** Stephanie Nougaret, Doenja M. J. Lambregts, Geerard L. Beets, Regina G. H. Beets-Tan, Lennart Blomqvist, David Burling, Quentin Denost, Maria A. Gambacorta, Benedetta Gui, Ann Klopp, Yulia Lakhman, Kate E. Maturen, Riccardo Manfredi, Iva Petkovska, Luca Russo, Atul B. Shinagare, James A. Stephenson, Damian Tolan, Aradhana M. Venkatesan, Aaron J. Quyn, Rosemarie Forstner

**Affiliations:** 1 Department of Radiology, PINKCC lab, U1194, Montpellier Cancer Center, Montpellier, France; 2 Department of Radiology, Netherlands Cancer Institute, Amsterdam, The Netherlands; 3 Department of Surgery, Netherlands Cancer Institute, Amsterdam, The Netherlands; 4 Department of Molecular Medicine and Surgery, Karolinska Institutet, Sweden & Department of Radiation Physics/Nuclear Medicine, Karolinska University Hospital, Solna, Sweden; 5 Intestinal Imaging Centre, St Mark’s Hospital, London North West University Healthcare NHS, London, UK; 6 Bordeaux ColoRectal institute, Clinique Tivoli, Bordeaux, France; 7 Department of Radiation Oncology, Fondazione Policlinico Universitario A.Gemelli IRCCS, Rome, Italy; 8 Department of Bioimaging, Radiation Oncology and Hematology, Fondazione Policlinico Universitario Agostino Gemelli IRCCS, Rome, Italy; 9 Department of Radiation Oncology, University of Texas M.D. Anderson Cancer Center, Houston, TX USA; 10 Department of Radiology, Memorial Sloan Kettering Cancer Center, New York, NY USA; 11 Departments of Radiology and Obstetrics and Gynecology, University of Michigan, Ann Arbor, MI USA; 12 Department of Bioimaging, Radiation Oncology and Hematology, UOC of Radiodiagnostica Presidio Columbus, Fondazione Policlinico Universitario A. Gemelli IRCSS, Rome, Italy; 13 Department of Radiology, Brigham and Women’s Hospital, Dana-Farber Cancer Institute, Harvard Medical School, Boston, MA USA; 14 Department of Radiology, University College London Hospitals NHS Foundation Trust, London, UK; 15 Department of Radiology, St James’s University Hospital, Leeds, UK; 16 Department of Abdominal Imaging, Division of Diagnostic Imaging, The University of Texas M.D. Anderson Cancer Center, Houston, TX USA; 17 John Goligher Colorectal Unit, Leeds Teaching Hospitals NHS Trust, Leeds, UK; 18 Department of Radiology, Uniklinikum Salzburg, PMU, Salzburg, Austria

**Correction to:** **European Radiology**

10.1007/s00330-024-10940-z published online 25 Aug 2024.

The article “Imaging in pelvic exenteration—a multidisciplinary practice guide from the ESGAR-SAR-ESUR-PelvEx collaborative group”, written by Stephanie Nougaret, Doenja M. J. Lambregts, Geerard L. Beets, Regina G. H. Beets-Tan, Lennart Blomqvist, David Burling, Quentin Denost, Maria A. Gambacorta, Benedetta Gui, Ann Klopp, Yulia Lakhman, Kate E. Maturen, Riccardo Manfredi, Iva Petkovska, Luca Russo, Atul B. Shinagare, James A. Stephenson, Damian Tolan, Aradhana M. Venkatesan, Aaron J. Quyn, Rosemarie Forstner, was originally published under exclusive license to The Author(s), under exclusive license to *European Society of Radiology*. As a result of the subsequent decision to publish the article under the open access model, the article’s copyright notice was changed on 22 January 2025 to © The Author(s) 2024 and the article is now distributed under a Creative Commons Attribution 4.0 International License (http://creativecommons.org/licenses/by/4.0/), which permits use, sharing, adaptation, distribution and reproduction in any medium or format, as long as you give appropriate credit to the original author(s) and the source, provide a link to the Creative Commons license and indicate if changes were made.

The images or other third-party material in this book are included in the book’s Creative Commons license, unless indicated otherwise in a credit line to the material. If material is not included in the book’s Creative Commons license and your intended use is not permitted by statutory regulation or exceeds the permitted use, you will need to obtain permission directly from the copyright holder.

