# Supplementary material for: Imaging in pelvic exenteration—a multidisciplinary practice guide from the ESGAR-SAR-ESUR-PelvEx collaborative group
Source: Eur Radiol. 2024 Aug 25;35(5):2681–91. doi: 10.1007/s00330-024-10940-z (PMC12021987; doi:10.1007/s00330-024-10940-z)
Supplement: Supplementary file 1 — Appendix A [file 330_2024_10940_MOESM1_ESM.pdf]

Appendix A

The search string for imaging-specific literature used the following key words combinations:

[(cervix) OR (cervical) OR (rectal) OR (colorectal) OR (prostate)) AND ((tumor) OR (cancer) OR (neoplasm)) AND ((pelvic exenteration) OR (LEER) OR (evisceration) OR (palliative surgery)) AND ((MRI) OR (magnetic resonance imaging) OR (CT) OR (computed tomography) OR (PET/CT) OR (Positron Emission Tomography/Computed Tomography)]

The literature search for non-specific imaging-related literature was conducted removing imaging modalities from the search string. In details, the following search string was used:
[(cervix) OR (cervical) OR (rectal) OR (colorectal) OR (prostate)) AND ((tumor) OR (cancer) OR (neoplasm)) AND ((pelvic exenteration) OR (LEER) OR (evisceration) OR (palliative surgery))
