# Supplementary material for: Imaging in pelvic exenteration—a multidisciplinary practice guide from the ESGAR-SAR-ESUR-PelvEx collaborative group
Source: Eur Radiol. 2024 Aug 25;35(5):2681–91. doi: 10.1007/s00330-024-10940-z (PMC12021987; doi:10.1007/s00330-024-10940-z)
Supplement: Supplementary file 2 — S1 [file 330_2024_10940_MOESM2_ESM.pdf]

a

REPORTING TEMPLATE

I – Tumor description (in case of multifocality, each lesion should be reported separately)

- Type: ☐ Primary tumour
☐ Recurrent tumour ▯ Type or recurrence: ☐ Local
☐ Lymph node
☐ Peritoneal
☐ Other: …………………
▯ Level of confidence: ☐ Indeterminate recurrence
☐ Likely recurrence
☐ Definite recurrence

- Location:
☐ Central compartment: rectum – incl. anastomosis if present, Douglas pouch, internal & external sphincter, levator ani muscle
☐ Anterior compartment: bladder, urethra, prostate & seminal vesicles, vas deferens, uterus, vagina fallopian tubes, pubic symphysis, superior & inferior ramus of pubic bone
☐ Posterior compartment: piriformis muscle, presacral fascia, sacrum, coccyx, sacrospinus & sacrotuberous ligaments, sciatic nerve & branches, coccygeus muscles
☐ Lateral compartment: ureters, obturator muscles, ischial spine, ischium, sacral nerves/roots, iliac arteries & veins, lateral pelvic lymph nodes

Further specification (prose description) of location: ……………………………………………..

- Size Longest dimension: …………. cm
Other dimensions (optional): …………. cm x …………. cm

II – Organs and structures with (potential) tumor involvement (BONVUE)*,**

- Organ/Structure 1: ………….
Based on: ☐ …. mm margin / ☐ focal contact / ☐ broad-based contact / ☐ frank invasion
Description: ………….

- Organ/Structure 2: ………….
Based on: ☐ …. mm margin / ☐ focal contact / ☐ broad-based contact / ☐ frank invasion
Description: ………….

- Organ/Structure 3, …, etc

* Note, in case of:
- **Vessel involvement:** describe longitudinal and radial extent of invasion, associated luminal narrowing, occlusion and/or deformity, and (optional) the presence of thrombus or collaterals
- **Ureter involvement:** describe longitudinal and radial extent of invasion, presence & degree of upstream dilatation, kidney function (hydronephrosis, cortical thinning, loss of enhancement)
- **Sacral nerve involvement:** describe which nerve roots are involved, and (optional) the distance (free margin) between the tumor and closest non-involved nerve root
- **Bone invasion**: describe whether there is superficial and/or deep bone invasion; in case of sacral bone invasion, also describe which vertebra are involved, and (optional) the distance from the sacral promontory and coccyx to the level of involvement
- **Anal canal & pelvic floor involvement**: describe which layers (internal sphincter, intersphincteric plane, external sphincter) are involved, and (optional) the craniocaudal extent of invasion

** BONVUE (Useful acronym to remember which structures to check and report):
Bones, **O**rgans, **N**erves**,** **V**essels, **U**reters, **E**xtra tumor sites

b

III – Additional tumor sites

- Associated lymph node metastases: ☐ No
☐ Yes, specify* …………………..
* incl. specified description of pelvic side wall nodes

- Extra-pelvic disease sites: ☐ No
☐ Yes, specify …………………..

IV – Reporting for restaging after neoadjuvant treatment

- Estimated degree of response: ☐ Progressive disease
☐ Stable disease
☐ Partial response
☐ Very good (incl. possible complete) response

- Reduction in size (longest diameter): From ….. cm on (date of prior study) to ….. cm

V – Other

- Relevant **anatomical variants** (incl. pelvic sidewall vessels)
- Description of pre-existent **post-treatment changes** (e.g. post-surgery or post-radiotherapy)
- (Optional: Surgical planning ‘roadmap’ discussion)
